# Synthesis, Characterization, and Catalytic Exploration of Mononuclear Mo(VI) Dioxido Complexes of (*Z*)-1-*R*-2-(4′,4′-Dimethyl-2′-oxazolin-2′-yl)-eth-1-en-1-ates †

**DOI:** 10.3390/molecules27041309

**Published:** 2022-02-15

**Authors:** Anna Petrov, Jeanette A. Adjei, Alan J. Lough, R. Stephen Wylie, Robert A. Gossage

**Affiliations:** 1 Department of Chemistry & Biology, Ryerson University, 350 Victoria Street, Toronto, ON M5B 2K3, Canada; annasophiajodhi@gmail.com (A.P.); jaadjei@ryerson.ca (J.A.A.); swylie@ryerson.ca (R.S.W.); 2 X-ray Crystallography Laboratory, University of Toronto, Toronto, ON M5S 3H6, Canada; alan.lough@utoronto.ca

**Keywords:** oxazoline-enolate chelates, molybdenum(6+) oxide complexes, X-ray structure determination, benzoin oxidation, semi-empirical PM6(tm) calculations

## Abstract

The coordination chemistry of the title ligands with Mo metal centers was investigated. Thus, the synthesis and characterization (NMR, X-ray diffraction) of four mononuclear formally Mo(6+) complexes of (*Z*)-1-*R*-2-(4′,4′-dimethyl-2′-oxazolin-2′-yl)-eth-1-en-1-ates (L: R = –Ph, –Ph-*p*-NO_2_, –Ph-*p*-OMe and –*t*-Bu), derived from the part enols (LH), is described. The resulting air-stable MoO_2_L_2_ complexes (**1**–**4**) exist, as shown by single-crystal X-ray diffraction experiments, in the *cis*-dioxido-*trans(N)*-κ^2^-*N*,*O*-L conformation in the solid state for all four examples. This situation was further probed using semi-empirical PM6(tm) calculations. Complexes **1**–**4** represent the first Mo complexes of this ligand class and, indeed, of Group 6 metals in general. Structural and spectroscopic comparisons were made between these and related Mo(6+) compounds. Complex **1** (R = –Ph) was studied for its ability to selectively catalyze the production of *poly*-norbornene from the monomer in the presence of MAO. This, unfortunately, only resulted in the synthesis of insoluble, presumably highly cross-linked, polymeric and/or oligomeric materials. However, complexes **1**–**4** were demonstrated to be highly effective for catalyzing benzoin to benzil conversion using DMSO as the *O*-transfer agent. This catalysis work is likewise put into perspective with respect to analogous Mo(6+) complexes.

## 1. Introduction

**Scheme 1:**
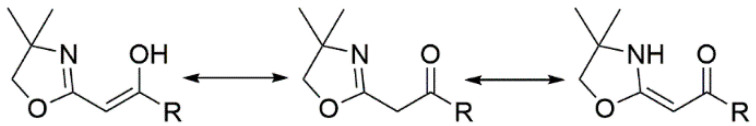
Three possible tautomers of *Tohda’s Ligands* (i.e., LH).

In recent years, we have been investigating the coordination chemistry and catalytic potential of metal and non-metal complexes derived from what we call *Tohda’s Ligands* (Scheme 1: LH). These ligand precursors were studied from an organic chemistry perspective for some time [1,2,3,4,5,6,7,8,9,10], particularly in the area of heterocyclic synthesis. The materials themselves can exist in at least three tautomers, i.e., two oxazoline forms, existing as an enol (Scheme 1, left) or keto isomer (Scheme 1: middle) or in the enamine heterocyclic configuration (Scheme 1, right). In the solid state, the latter of these is the predominant isomer [7,9], with the keto isomer sometimes being observed in solution depending on “R”, the solvent(s), and other factors (e.g., protonation at *N*) [1,2,3,4,5,6,7,8,9,10]. 

Our primary interest in these materials stems from their potential to act as ligands, either in a monomeric sense, likely solely via *N*(oxazoline) binding, or by chelation (or bridging) bonding motifs involving the same *N*-atom and the non-heterocyclic-*O*. This latter atom can potentially bind in a neutral sense or anionically following deprotonation of the LH unit. Thus far, we have only observed the κ^2^-*N*,*O* bonding motif following formal H^+^ loss from LH; hence, metal-bound enolate anions (L^−^) are found within the metal coordination sphere. This has been shown in the chemistry of Co^2+/3+^, Cu^2+^ [9,11], Ni^2+^ [12], and Ir^+/3+^ [13], in addition to main group examples [14]. Herein, we detail our exploration into the reactivity of formally Mo^6+^ metal centers with LH derivatives, as the reactivity of these compounds with Group 6 metals is currently completely unexplored. In this regard, several examples with general formula MoO_2_(L)_2_ will be described in addition to some preliminary experiments probing the catalytic potential of these new complexes. This *Article* is part of a special issue of *Molecules* in celebration of the 80th birthday of *Prof. Gerard van Koten* [15].

## 2. Results

### 2.1. Synthesis and Characterisation

**Scheme 2:**
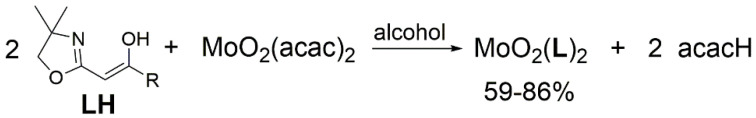
General synthesis methodology to yield complexes **1**–**4** (**1**: R = –Ph; **2**: R = –Ph-p-NO_2_; **3**: R = –Ph-p-OMe; **4**: R = –t-Bu).

The synthesis of the (*Z*)-1-*R*-2-(4′,4′-dimethyl-2′-oxazolin-2′-yl)-eth-1-en-1-ols used here (L^1^H: R = –Ph; L^2^H: R = –Ph-*p*-NO_2_; L^3^H: R = –Ph-*p*-OMe; L^4^H: R = –t-Bu: Scheme 2) was carried out as previously described [2,9,16]. The treatment of alcoholic solutions (L^n^H; *n* = 1, 2 and 4: MeOH; L^3^H: 95% aq. EtOH) of LH (~2 equivalents) with *cis*-MoO_2_(acac)_2_ (acac = κ^2^-*O*,*O*’-acetylacetonato) [17,18,19] leads to the facile formation of complexes **1**–**4**, presumably involving the loss of acacH (Scheme 2), in good to moderate yields (see Section 4).

**Scheme 3:**
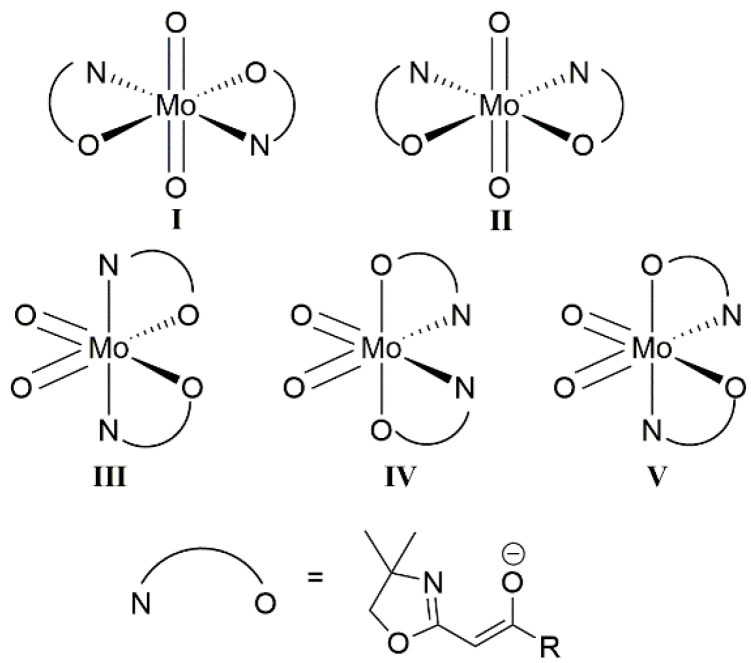
The five possible structural isomers of a hypothetical unit of MoO_2_(L)_2_.

Complexes **1**–**4** are air- and moisture-stable solids and range in color from yellow-orange to orange to red. Elemental analysis of all four species was consistent with materials having the empirical composition of MoO_2_(L)_2_. Spectroscopic characterization (^1^H and ^13^C) revealed a situation in which both the methyl and the methylene protons of the oxazoline ring of L were in a non-symmetrical environment, and hence diastereotopic H atoms were noted. This suggested a lack of mirror plane symmetry with respect to these units. Hence, the all-trans isomer and the trans-dioxido-cis-bis-κ^2^-*N*,*O*-L isomers of a hypothetical mononuclear octahedral Mo^6+^ complex were unlikely structural variants (Scheme 3: **I** and **II**). However, the above NMR studies could not confirm either the mononuclear nature of these materials (i.e., [MoO_2_(L)_2_]_n_; *n* = 1) or the most stable of the cis-MoO_2_ isomer(s) (Scheme 3: **III**–**V**). We therefore approached these isomeric possibilities using a combination of X-ray diffraction studies and semi-empirical PM6(tm) calculations. This latter technique has been found useful for relatively complex systems in terms of approximating the relative stability of various isomeric forms of transition metal complexes [20,21].

### 2.2. Semi-Empirical PM6(tm) Treatment of Hypothetical Complex **5**

A semi-empirical treatment at the PM6(tm) level of theory was initially used to gain some insight into the likely ligand disposition around the MoO_2_(L)_2_ unit [21]. For computational simplicity, a hypothetical complex in which R = –CH_3_ (Scheme 2: complex **5**) was used for the five possible structural forms (Scheme 3: **I**–**V**). The PM6(tm) results clearly suggested (see Section 4.3) that the two trans-dioxido isomers **I** and **II** were indeed energetically unfavorable with respect to the cis-forms, as attempts to model these led to convergence at the cis-isomers. This observation is in agreement with the spectroscopic characterization of the isolated complexes **1**–**4**. Hence, it is likely that **1**–**4** retain the cis-orientation of the two Mo=O units known to be present in the starting complex MoO_2_(acac)_2_ [17,18,19]. Of the cis-dioxido isomers, form **III** (trans-N_ox_: ox = oxazoline) was found to be the most stable (relative values of ΔH_f_ = 0.00; +56.8 and +44.5 kJ mol^–1^ respectively, for **III**, **IV** and **V**). As both the level of theory and the adjusted R group on L may inadvertently skew these results, we took to employing solid-state X-ray diffraction studies of complexes **1**–**4** to unequivocally solve this isomeric dilemma.

### 2.3. X-ray Structural Studies

**Scheme 4:**
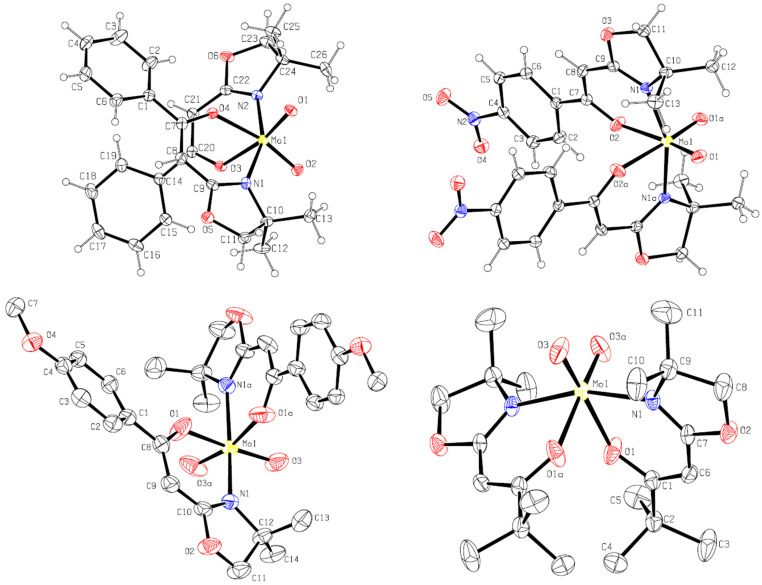
ORTEP representations of unit cell molecules of **1** (**top**, **left**), **2** (**top**, **right**), **3** (**bottom**, **left**), and **4** (**bottom**, **right**). Thermal ellipsoids are at the 30% calculated level.

Suitable crystalline material of the complexes described herein were obtained by the solvent layering technique using either CHCl_3_ (**1**, **3** and **4**) or acetone (**2**) solutions of the complex that had been thereafter layered with hexanes and then left sealed and undisturbed for several hours or days to induce crystal formation. ORTEP representations [22] of a unit cell molecule of each complex are displayed in Scheme 4. Table 1 below reports the observed crystal parameters for the solid-state characterization of **1**–**4**, and Table 2 presents selected bond lengths and angles for the four complexes [23,24].

These data (Table 1 and Table 2 and Supplementary Information) confirmed the presumed mononuclear nature, i.e., [MoO_2_(L)_2_]_n_: *n* = 1, of all four complexes described. As anticipated from the computational evaluation, the complexes adopted a distorted octahedral ligand atom arrangement around the Mo center in all cases. A *cisoidal* orientation of the oxido ligands was observed with *trans*-spanning imine *N*-atoms of the oxazoline units, thus requiring a *cisoidal* positioning of the remaining, formally enolate, *O*-donor atoms. These results were fully consistent with the PM6(tm) treatment.

### 2.4. Catalytic Explorations

#### 2.4.1. Norbornene Polymerization

**Scheme 5:**
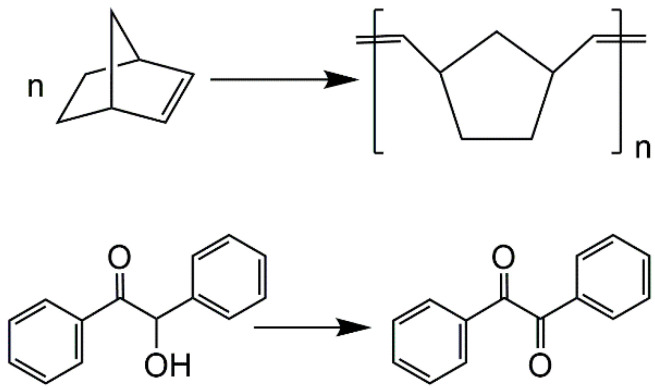
Schematic representations of the ROMP of norbornene to yield poly-norbornene (**top**) and oxidation of benzoin to benzil (**bottom**).

Compound **1** was tested for its potential as a promoter of the ring-opening metathesis polymerization (ROMP) of norbornene as the test substrate (Scheme 5: top). Structurally related Mo^6+^ complexes are known to be effective at such polymerization in the presence of alkyl-aluminum promoters such as methylaluminoxane (MAO). Thus, complex **1** in catalytic proportions (10 mol%) was exposed to a solution of norbornene with excess MAO under typical polymerization conditions (see Section 4.4.1). Although polymerization was noted, the resulting polymeric and/or oligomeric matrix was found to be completely insoluble in any tested solvents. It was assumed that highly cross-linked *poly*-norbornene was the likely product [25], and thus further attempts to optimize this process were abandoned.

#### 2.4.2. Benzoin Oxidation to Benzil

Complexes **1**–**4** were also tested for their ability to initiate O-transfer chemistry. The catalytic oxidation of benzoin to benzil is a well-known benchmark reaction for high-valent Group 6 dioxido complexes; dimethylsulfoxide (DMSO) is a typical agent used to transfer the oxygen atom [26]. Unlike the polymerization experiments described above, all four catalysts gave clean conversion (Section 4.4.2 [26]) of benzoin to benzil using DMSO at elevated temperatures (Scheme 5: bottom). Somewhat surprisingly, complex **1** (R = –Ph) and **4** (R = –t-Bu) resulted in essentially quantitative conversion; however, **2** and **3**, containing the electron-withdrawing –NO_2_ group or the electron-donating –OMe group, respectively, yielded slightly lower conversions (80–87%) under identical conditions. Note that these reactions were performed as described (Section 4.4.2) to simply explore any potential activity; no attempt was made to optimize these conditions specifically for this system.

## 3. Discussion

### 3.1. Synthesis and Structural Investigations

Complexes **1**–**4** represent the first Group 6 compounds incorporating metal-bound enolates derived from the LH ligand set. The straightforward reaction of readily available *cis*-MoO_2_(acac)_2_ to give the desired air-stable materials occurred in the absence of an added base, a situation that is typically required to produce reaction conditions necessary to coordinate the enolate fragment obtained from LH. Hence, these Mo^6+^-based materials formed more readily when compared to most of the Ni^2+^, Co^2+^, Cu^2+^, and Ir^+^ complexes reported previously [9,11,12,13]. Molecular modeling (Section 2.2) suggested that the likely ligand orientation around a mononuclear Mo^6+^ center was that of isomer **III** (Scheme 3). A confirmation of that concept was obtained for all four complexes, regardless of the “R” group functionality on the ene-scaffold (Scheme 2), as revealed by the X-ray diffraction studies. The structural results support the notion that the use of relatively simple PM6(tm) semi-empirical calculations to probe relative isomer stability in such systems is effective and quite computationally undemanding [20,21]. Surprisingly, little structural diversity of complexes **1**–**4** was noted in terms of ligand framework or ligated atoms around each Mo atom (Table 2; Supplementary Information). The *cis*-orientation of the dioxido unit was confirmed in addition to the presence of *trans*-spanning *N* atoms and *cis*-enolate *O*-groups. The bond lengths between the metal and these atoms can be compared to structurally related examples and are otherwise typical [23,24,25,26,27,28,29,30,31,32,33,34,35,36,37,38,39,40,41]. These data did not show large differences in magnitude throughout the series. The main observed variation involved the N–Mo–N bond angle, which varied from ~166° in **3** to ~158° in **1**.

### 3.2. Catalysis

High-valent Group 6 oxo complexes are well known to be active complexes for the catalytic ROMP of olefins [42,43,44,45,46,47]. Unfortunately, our study of complex **1** in this regard did not yield conclusive results concerning the nature of the polymers derived from norbornene under typical conditions. Indeed, completely insoluble and presumably highly cross-linked products (Section 2.4.1) were isolated [25]. In contrast, complexes **1**–**4** were useful in oxygen-transfer chemistry (Section 2.4.2), and the activity of these materials parallels similar systems [26,27,28,29,30,31,32,33,34,35,36,37,38,39,40,41,48] in the benchmark formation of benzil from benzoin (Scheme 5).

## 4. Materials and Methods

### 4.1. Synthesis

#### 4.1.1. General Information

All reactions and purifications were performed under ambient conditions. Solvents were dried/purified using an mBraun Solvent Purification System (SPS). All reagent chemicals and solvents used herein were purchased commercially. NMR experiments were performed with a Bruker AV 400 Spectrometer at ambient temperature. All NMR spectral data are reported in values of δ against external TMS (δ = 0.00 ppm: ^1^H, ^13^C). Abbreviations for NMR experimental line listings are as follows: s—singlet; d—doublet; m—multiplet. Uncorrected melting points were obtained using a Fisher Scientific melting point apparatus. UV–Vis. spectra were recorded from CH_2_Cl_2_ solutions using an Agilent Cary 5000 spectrometer with a 1 cm-path length cuvette. IR spectra were recorded in the solid state using an Agilent Cary 630 FTIR spectrometer. Compounds L^1^H–L^4^H were synthesized as previously reported [2,9,16].

#### 4.1.2. Complex **1**: MoO_2_(κ^2^-*N*,*O*-L^1^)_2_

A sample of L^1^H (0.50 g: 2.3 mmol) was dissolved in 5 mL of MeOH. To this solution MoO_2_(acac)_2_ (0.36 g; 1.1 mmol) was added, and the mixture was stirred at room temperature (RT) for 6 h. During this time period, a bright orange-colored precipitate formed. This material was filtered off, washed with further MeOH (3 mL), and then allowed to dry under ambient conditions. The yield of this material, complex 1, was 0.49 g (75%). Crystals of 1 suitable for X-ray diffraction were obtained by the slow mixing of a CHCl_3_ solution of 1 that had been layered with hexanes. Mp = 219–220 °C (decomp.); selected IR (υ): 1524, 961, 881, 758 cm^−1^; UV-Vis. (λ_max_ [log ε]; 3.45 × 10^−2^ M): 284 [4.42], 326 [4.27], 393 [3.78] nm; ^1^H-NMR (400 MHz, CDCl_3_): δ = 1.67 (s, 6H, -CH_3_), 1.73 (s, 6H, -C*H*_3_), 4.05 (d, 2H, *J* = 8.0 Hz, -C*H*_2_), 4.28 (d, 2H, *J* = 8.0 Hz, -C*H*_2_), 5.56 (s, 2H, =C*H*), 7.24–7.38 and 7.57–7.59 (m, 10H Ar*H*); ^13^C{^1^H}-NMR (101 MHz, CDCl_3_): δ = 26.8, 27.5, 68.9, 79.3, 83.2, 126.8, 128.0, 130.5, 137.2, 171.3, 177.7; elemental analysis calcd (%) for C_26_H_28_N_2_O_6_Mo (560.42): C 55.72, H 5.04, N 5.00; found: C 55.96, H 5.31, N 4.96. 

#### 4.1.3. Complex **2**: MoO_2_(κ^2^-*N*,*O*-L^2^)_2_

A sample of L^2^H (0.50 g: 1.9 mmol) was dissolved in 5 mL of MeOH. To this solution MoO_2_(acac)_2_ (0.31 g; 1.0 mmol) was added, and the mixture was heated to reflux temperature for 20 h. During this time period, a yellow-orange-colored precipitate formed. This material was filtered off, washed with further MeOH (3 mL), and then allowed to dry under ambient conditions. The yield of this material, complex 2, was 0.50 g (76%). Crystals of 2 suitable for X-ray diffraction were obtained by the slow mixing of a CHCl_3_ solution of 2 that had been layered with hexanes. Mp = >230 °C; selected IR (υ): 1512, 971, 747 cm^−1^; UV-Vis. (λ_max_ [log ε]; 2.63 × 10^−2^ M): 258 [4.22], 266 [4.57] nm; ^1^H-NMR (400 MHz, CDCl_3_): δ = 1.66 (s, 6H, -CH_3_), 1.74 (s, 6H, -C*H*_3_), 4.12 (d, 2H, *J* = 8.0 Hz, -C*H*_2_), 4.36 (d, 2H, *J* = 8.0 Hz, -C*H*_2_), 5.58 (s, 2H, =C*H*), 7.70 (d, *J* = 8.8 Hz, 4H Ar*H*), 8.14 (d, *J* = 8.8 Hz, 4H Ar*H*); ^13^C{^1^H}-NMR (101 MHz, CDCl_3_): δ = 26.7, 27.4, 69.3, 79.6, 85.1, 123.5, 127.4, 142.7, 148.9, 171.1, 174.2; elemental analysis calcd (%) for C_26_H_26_N_4_O_10_Mo (622.40): C 48.01, H 4.03, N 8.61; found: C 48.14, H 4.05, N 8.54.

#### 4.1.4. Complex **3**: MoO_2_(κ^2^-*N*,*O*-L^3^)_2_

A sample of L^3^H (0.50 g: 2.0 mmol) was dissolved in 5 mL of 95% aq. EtOH. To this solution MoO_2_(acac)_2_ (0.33 g; 1.0 mmol) was added, and the mixture was stirred at RT for 20 h. During this time period, a red-colored precipitate had formed. This material was filtered off, washed with further EtOH (3 mL), and then allowed to dry under ambient conditions. The yield of this material, complex 3, was 0.54 g (86%). Crystals of 3 suitable for X-ray diffraction were obtained by the slow mixing of an acetone solution of 3 that had been layered with hexanes. Mp = 201–203 °C; selected IR (υ): 1532, 882, 778 cm^−1^; UV-Vis. (λ_max_ [log ε]; 3.43 × 10^−2^ M): 297 [4.52], 313 [4.96], 378 [3.91] nm; ^1^H-NMR (400 MHz, CDCl_3_): δ = 1.66 (s, 6H, -C*H*_3_), 1.72 (s, 6H, -C*H*_3_), 3.79 (s, 6H, -OC*H*_3_), 4.02 (d, 2H, *J* = 8.0 Hz, -C*H*_2_), 4.28 (d, 2H, *J* = 8.0 Hz, -C*H*_2_), 5.55 (s, 2H, =C*H*), 6.77 (d, *J* = 8.8 Hz, 4H Ar*H*), 7.55 (d, *J* = 8.8 Hz, 4H Ar*H*); ^13^C{^1^H}-NMR (101 MHz, CDCl_3_): δ = 26.7, 27.4, 55.3, 68.8, 79.2, 82.0, 113.2, 128.6, 129.8, 161.5, 171.2, 177.5; elemental analysis calcd (%) for C_28_H_32_N_2_O_8_Mo (620.47): C 54.20, H 5.20, N 4.51; found: C 54.38, H 5.15, N 4.46.

#### 4.1.5. Complex **4**: MoO_2_(κ^2^-*N*,*O*-L^4^)_2_

A sample of L^4^H (0.50 g: 2.5 mmol) was dissolved in 5 mL of MeOH. To this solution MoO_2_(acac)_2_ (0.41 g; 1.3 mmol) was added, and the mixture was stirred at RT for 3 h. During this time period, an orange-colored precipitate formed. This material was filtered off, washed with further MeOH (3 mL), and then allowed to dry under ambient conditions. The yield of this material, complex 4, was 0.39 g (59%). Crystals of 4 suitable for X-ray diffraction were obtained by the slow mixing of a CHCl_3_ solution of 4 that had been layered with hexanes. Mp = 180–182 °C; selected IR (υ): 1533, 870, 774 cm^−1^; UV-Vis. (λ_max_ [log ε]; 3.54 × 10^−2^ M): 266 [4.28], 308 [3.86], 434 [2.99] nm; ^1^H-NMR (400 MHz, CDCl_3_): δ = 1.06 (s, 18H, -C*H*_3_), 1.57 (s, 6H, -C*H*_3_), 1.64 (s, 6H, -C*H*_3_), 3.94 (d, 2H, *J* = 8.0 Hz, -C*H*_2_), 4.13 (d, 2H, *J* = 8.0 Hz, -C*H*_2_), 5.10 (s, 2H, =C*H*); ^13^C{^1^H}-NMR (101 MHz, CDCl_3_): δ = 27.0, 27.6, 27.9, 39.3, 68.6, 79.0, 80.6, 171.3, 192.8; elemental analysis calcd (%) for C_22_H_36_N_2_O_6_Mo (520.44): C 50.77, H 6.97, N 5.38; found: C 49.35, H 6.43, N 5.08. Note that the obtained C% was found to be outside the typical acceptable limits.

### 4.2. Semi-Empirical PM6(tm) Calculations

Calculations at the PM6(tm) level of theory were performed using the Gaussian 16.0 suite of software [49]. Vibrational energy calculations revealed no imaginary frequencies, and hence a stable conformation on the potential energy surface was assumed.

### 4.3. X-ray Diffraction

X-ray diffraction data were collected on a Nonius Kappa CCD diffractometer using Mo Kα radiation (λ = 0.71073 Å) obtained using Collect [50]. Cell refinement and data reduction were performed with Denzo–SMN [51]. The structure solution employed SIR-92 [52], and the structure refinement was carried out with SHELXL [53]. The molecular graphics were obtained using PLATON [54].

### 4.4. Catalysis

#### 4.4.1. Norbornene Polymerization

Norbornene (5.76 g: 61.2 mmol), toluene (5 mL), and 1 (0.67 g: 1.2 mmol) were mixed together in a flame-dried Schlenk flash under an N_2_ (*g*) atmosphere. To the mixture under stirring MAO (2.0 M toluene; 1.2 mL: 2.4 mmol) was added, and the resulting mixture was stirred for 5 h at RT. The color of the mixture gradually changed from orange to brown to eventually a shade of off purple. The solid mass that resulted from this process, which eventually became so viscous that it prevented further stirring, was found to be completely insoluble in all tested solvents. 

#### 4.4.2. Benzoin Oxidation

In a typical reaction, 21 mg of benzoin (0.10 mmol), 0.01 mmol of catalyst (10 mol%), and an internal standard (30 mg C_6_Me_6_) were dissolved in 0.7 mL of deoxygenated d_6_-dmso in an NMR tube. The tube was heated to 120 °C for 24 h and then cooled to RT. The mixture was then diluted with 0.7 mL of CDCl_3_, and the yield of benzin was thereafter determined by ^1^H NMR spectroscopy [32,33,36,37,40,41,48]. 

## 5. Conclusions

The first examples of Group 6 metal complexes of the title ligands were reported, represented by a series of MoO_2_L_2_ derivatives. These materials appeared to be active catalysts in *O*-atom transfer chemistry. Overall, the transition metal chemistry of *Tohda’s Ligands*, i.e., (*Z*)-1-*R*-2-(4′,4′-dimethyl-2′-oxazolin-2′-yl)-eth-1-en-1-ols, continues to yield rich coordination chemistry and catalytic potential. Future studies will continue this exploration including both late and early transition metal chemistry, in addition to the lanthanide elements. 

## Figures and Tables

**Table 1:** Crystallographic parameters for the solid-state characterization of complexes **1**–**4** ^a^.

Data	Complex 1	Complex 2	Complex 3	Complex 4
Empirical Formula	C_26_H_38_N_2_O_6_Mo	C_26_H_26_N_4_O_10_Mo	C_28_H_32_N_2_O_8_Mo	C_22_H_36_N_2_O_6_Mo
Formula Weight (g/mol)	560.44	650.45	620.50	520.47
Temperature (K)	150(2)	150(2)	150(2)	150(2)
Wavelength (Å)	0.71073	0.71073	0.71073	0.71073
Crystal System	monoclinic	monoclinic	orthorhombic	trigonal
Space group	*P* 21/n	*C* 2/c	*P* b c n	P$\bar{3}$c1
Unit Cell Dimensions (Å)	*a* = 11.1664(4)	*a* = 20.5793(6)	*a* =11.2202(2)	*a* = 19.9342(10)
(Å)	*b* = 11.1623(4)	*b* = 8.1384(3)	*b* = 12.9081(3)	*b* = 19.9342(10)
(Å)	*c* = 20.4516(4)	*c* = 17.5927(6)	*c* = 18.7510(4)	*c* = 11.5030(4)
(°)	α = 90	α = 90	α = 90	α = 90
(°)	β = 95.6150(19)	β = 113.6580(18)	β = 90	β = 90
(°)	γ = 90	γ = 90	γ = 90	γ = 120
Volume (Å^3^)	2563.91(14)	2698.84(16)	2715.73(10)	3958.6(3)
*Z*	4	4	4	6
Density (calcd; mg/m^3^)	1.467	1.601	1.518	1.310
Absorp. Coefficient (mm^−1^)	0.560	0.551	0.536	0.532
*F*(000)	1152	1328	1280	1632
Crystal Size (mm^3^)	0.40 × 0.30 × 0.22	0.20 × 0.18 × 0.16	0.18 × 0.14 × 0.12	0.20 × 0.20 × 0.10
θ range for Data Collection (°)	2.59–27.48	2.58–27.51	2.64–27.48	2.70–27.48
Index Ranges	−14 ≤ *h* ≤ 14	−26 ≤ *h* ≤ 23	−14 ≤ *h* ≤ 14	−22 ≤ *h* ≤ 22
	−14 ≤ *k* ≤ 14	−10 ≤ *k* ≤ 10	−16 ≤ *k* ≤ 16	−25 ≤ *k* ≤ 25
	−21 ≤ *l* ≤ 26	−22 ≤ *l* ≤ 22	−24 ≤ *l* ≤ 24	0 ≤ *l* ≤ 14
Reflexions Collected	16,606	12,858	19,994	3021
Independent Reflexions	5765 [R(int) = 0.0492]	3089 [R(int) = 0.0391]	3120 [R(int) = 0.0455]	3021 [R(int) = 0.049]
Completeness to θ = *x*° (%)	*x* = 25.25 (99.6)	*x* = 27.51 (99.3)	*x* = 27.48 (99.9)	*x* = 27.48 (99.5)
Absorption Correction	Semi-empirical from equivalents	Semi-empirical from equivalents	Semi-empirical from equivalents	Semi-empirical from equivalents
Max./min. Transmission	0.888/0.752	0.917/0.851	0.770/0.629	0.949/0.897
Refinement Method	Full-matrix least squares on *F*^2^	Full-matrix least squares on *F*^2^	Full-matrix least squares on *F*^2^	Full-matrix least squares on *F*^2^
Data/Restraints/Parameters	5765/0/320	3089/0/188	3120/0/180	3021/0/146
Goodness of Fit on *F*^2^	1.069	1.107	1.071	1.041
Final *R* indices [*I* > 2σ(*I*)]	*R*1 = 0.0441; w*R*2 = 0.0917	*R*1 = 0.0404; w*R*2 = 0.0949	*R*1 = 0.0449; w*R*2 = 0.1142	*R*1 = 0.0522; w*R*2 = 0.1352
*R* indices (all data)	*R*1 = 0.0689; w*R*2 = 0.1034	*R*1 = 0.0521; w*R*2 = 0.1022	*R*1 = 0.0702; w*R*2 = 0.1339	*R*1 = 0.0852; w*R*2 = 0.1511
Largest diff. peak and hole (eÅ^−3^)	1.525 and 0.816	1.715 and 0.784	2.105 ad 0.975	1.437 and −0.742

^a^ Estimated standard deviations for the measured parameters are in parentheses.

**Table 2:** Selected bond lengths (Å°) and angles (°) for complexes **1**–**4** ^a^.

**Observed Data**	**Complex 1**	**Complex 2**	**Complex 3**	**Complex 4**
Mo=O	1.705(2); 1.709(2)	1.700(2)	1.690(3)	1.697(3)
Mo-O	2.101(2); 2.119(2)	2.103(2)	2.096(2)	2.072(3)
Mo-N	2.117(3); 2.135(3)	2.150(2)	2.138(3)	2.147(3)
C=N	1.331(4); 1.314(4)	1.310(4)	1.324(4)	1.311(4)
C=C	1.369(5); 1.373(5)	1.370(4)	1.371(5)	1.351(5)
O=Mo=O	102.10(11)	102.39(16)	104.3(2)	101.5(2)
N-Mo-N	158.37(10)	159.75(12)	166.28(14)	162.96(16)
O-Mo-O	80.08(9)	79.44(13)	77.41(19)	78.9(2)

^a^ Estimated standard deviations for the measured parameters are in parentheses; C=N refers to the formal C=N bond of the oxazoline group(s); C=C refers to the formal C=C of the C–C=C–O unit.

## Data Availability

All data are contained within the article and the Supplementary Materials.

## Supplementary Materials

The following are available online, Tables S1–S45: Full X-ray structural datasets for **1**–**4**, relevant .mol files for isomers **I**–**V**. CCDC 2131173-2131176 also contains supplementary crystallographic data for this paper. These data can be obtained free of charge from The Cambridge Crystallographic Data Centre via www.ccdc.cam.ac.uk/structures (accessed on 26 December 2021).

## Sample Availability

Samples of the compounds are available for purchase from the authors.
