# Supplementary material for: Synthesis, Characterization, and Catalytic Exploration of Mononuclear Mo(VI) Dioxido Complexes of (Z)-1-R-2-(4′,4′-Dimethyl-2′-oxazolin-2′-yl)-eth-1-en-1-ates
Source: Molecules. 2022 Feb 15;27(4):1309. doi: 10.3390/molecules27041309 (PMC8874378; doi:10.3390/molecules27041309)

checkCIF/PLATON report

Structure factors have been supplied for datablock(s) k09179

THIS REPORT IS FOR GUIDANCE ONLY. IF USED AS PART OF A REVIEW PROCEDURE FOR PUBLICATION, IT SHOULD NOT REPLACE THE EXPERTISE OF AN EXPERIENCED CRYSTALLOGRAPHIC REFEREE.

No syntax errors found.   CIF dictionary   Interpreting this report

Datablock: k09179

Bond precision:	C-C = 0.0053 A		Wavelength=0.71073
Cell:	a=11.1664(4)	b=11.1623(4)	c=20.4516(4)
	alpha=90	beta=95.6150(19)	gamma=90
Temperature:	150 K		

	Calculated	Reported
Volume	2536.91(14)	2536.91(14)
Space group	P 21/n	P 21/n
Hall group	-P 2yn	-P 2yn
Moiety formula	C26 H28 Mo N2 O6	C26 H28 Mo N2 O6
Sum formula	C26 H28 Mo N2 O6	C26 H28 Mo N2 O6
Mr	560.44	560.44
Dx,g cm-3	1.467	1.467
Z	4	4
Mu (mm-1)	0.560	0.560
F000	1152.0	1152.0
F000'	1145.60	
h,k,lmax	14,14,26	14,14,26
Nref	5833	5765
Tmin,Tmax	0.817,0.884	0.752,0.888
Tmin'	0.799	

Correction method= # Reported T Limits: Tmin=0.752 Tmax=0.888
AbsCorr = MULTI-SCAN

Data completeness= 0.988   Theta(max)= 27.477

R(reflections)= 0.0441(4415)   wR2(reflections)= 0.1034(5765)

S = 1.069   Npar= 320

```
The following ALERTS were generated. Each ALERT has the format
       test-name_ALERT_alert-type_alert-level.
Click on the hyperlinks for more details of the test.
```

Alert level C

```
PLAT601_ALERT_2_C Unit Cell Contains Solvent Accessible VOIDS of .         54 Ang**3
PLAT906_ALERT_3_C Large K Value in the Analysis of Variance ......      2.242 Check
PLAT911_ALERT_3_C Missing FCF Refl Between Thmin & STh/L=    0.600         14 Report
```

Alert level G

```
PLAT153_ALERT_1_G The s.u.'s on the Cell Axes   are Equal ..(Note)     0.0004 Ang.
PLAT398_ALERT_2_G Deviating  C-O-C    Angle From 120 for O5             107.4 Degree
PLAT398_ALERT_2_G Deviating  C-O-C    Angle From 120 for O6             106.1 Degree
PLAT794_ALERT_5_G Tentative Bond Valency for Mo1       (VI)      .       6.05 Info
PLAT910_ALERT_3_G Missing # of FCF Reflection(s) Below Theta(Min).          4 Note
PLAT912_ALERT_4_G Missing # of FCF Reflections Above STh/L=  0.600         51 Note
PLAT913_ALERT_3_G Missing # of Very Strong Reflections in FCF ....          1 Note
PLAT941_ALERT_3_G Average HKL Measurement Multiplicity ..........         2.9 Low
PLAT978_ALERT_2_G Number C-C Bonds with Positive Residual Density.          2 Info
```

```
   0 ALERT level A = Most likely a serious problem - resolve or explain
   0 ALERT level B = A potentially serious problem, consider carefully
   3 ALERT level C = Check. Ensure it is not caused by an omission or oversight
   9 ALERT level G = General information/check it is not something unexpected

   1 ALERT type 1 CIF construction/syntax error, inconsistent or missing data
   4 ALERT type 2 Indicator that the structure model may be wrong or deficient
   5 ALERT type 3 Indicator that the structure quality may be low
   1 ALERT type 4 Improvement, methodology, query or suggestion
   1 ALERT type 5 Informative message, check
```

Validation response form

Please find below a validation response form (VRF) that can be filled in and pasted into your CIF.

```
# start Validation Reply Form
_vrf_PLAT601_k09179
;
PROBLEM: Unit Cell Contains Solvent Accessible VOIDS of .         54 Ang**3
RESPONSE: ...
;
_vrf_PLAT906_k09179
;
PROBLEM: Large K Value in the Analysis of Variance ......      2.242 Check
RESPONSE: ...
;
_vrf_PLAT911_k09179
;
```

```
PROBLEM: Missing FCF Refl Between Thmin & STh/L=    0.600         14 Report
RESPONSE: ...
;
# end Validation Reply Form
```

It is advisable to attempt to resolve as many as possible of the alerts in all categories. Often the minor alerts point to easily fixed oversights, errors and omissions in your CIF or refinement strategy, so attention to these fine details can be worthwhile. In order to resolve some of the more serious problems it may be necessary to carry out additional measurements or structure refinements. However, the purpose of your study may justify the reported deviations and the more serious of these should normally be commented upon in the discussion or experimental section of a paper or in the "special_details" fields of the CIF. checkCIF was carefully designed to identify outliers and unusual parameters, but every test has its limitations and alerts that are not important in a particular case may appear. Conversely, the absence of alerts does not guarantee there are no aspects of the results needing attention. It is up to the individual to critically assess their own results and, if necessary, seek expert advice.

Publication of your CIF in IUCr journals

A basic structural check has been run on your CIF. These basic checks will be run on all CIFs submitted for publication in IUCr journals (*Acta Crystallographica*, *Journal of Applied Crystallography*, *Journal of Synchrotron Radiation*); however, if you intend to submit to *Acta Crystallographica Section C* or *E* or *IUCrData*, you should make sure that full publication checks are run on the final version of your CIF prior to submission.

Publication of your CIF in other journals

Please refer to the *Notes for Authors* of the relevant journal for any special instructions relating to CIF submission.

PLATON version of 18/12/2021; check.def file version of 18/12/2021

Datablock k09179 - ellipsoid plot

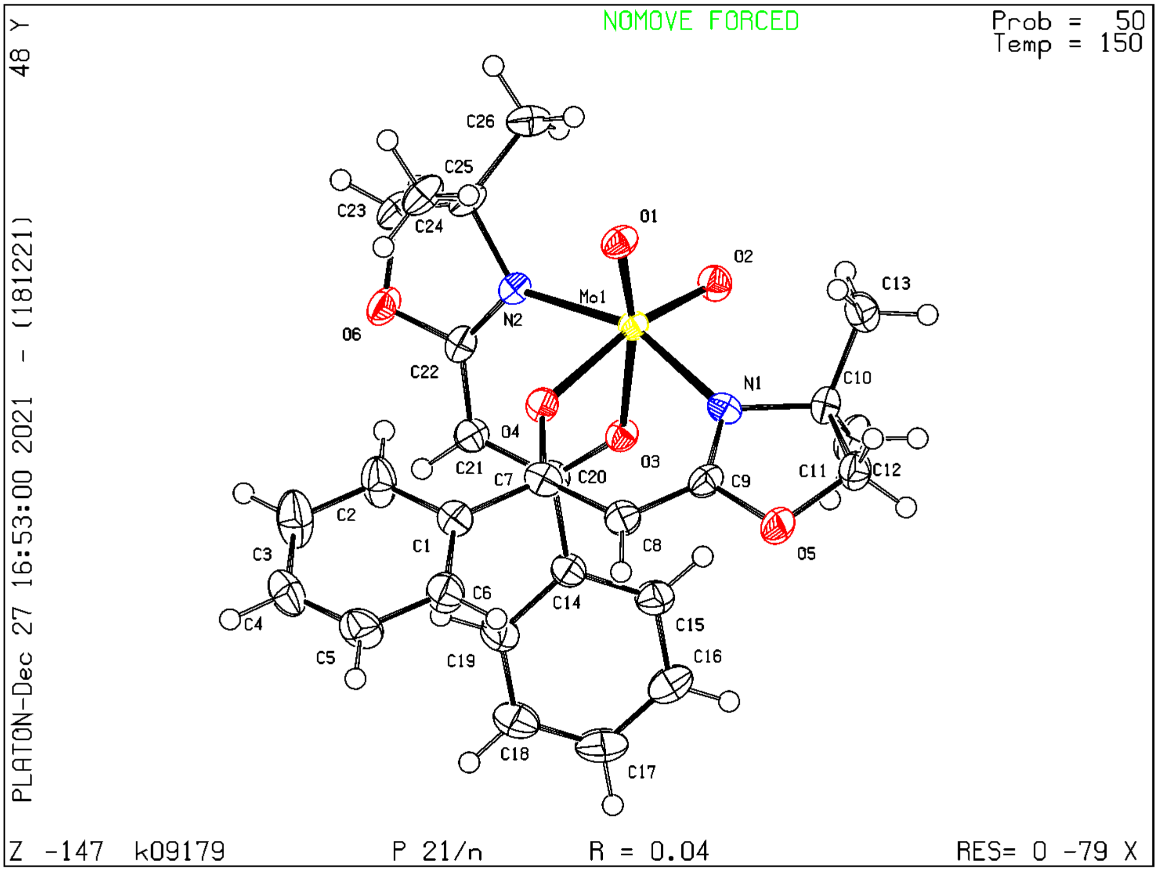

Supplement: Supplementary file 1 [file molecules-27-01309-s001.zip › molecules-1551548-supplementary/ESI-zipped/checkcif_2131173.pdf]
