# Supplementary material for: Synthesis, Characterization, and Catalytic Exploration of Mononuclear Mo(VI) Dioxido Complexes of (Z)-1-R-2-(4′,4′-Dimethyl-2′-oxazolin-2′-yl)-eth-1-en-1-ates
Source: Molecules. 2022 Feb 15;27(4):1309. doi: 10.3390/molecules27041309 (PMC8874378; doi:10.3390/molecules27041309)

checkCIF/PLATON report

Structure factors have been supplied for datablock(s) k09188

THIS REPORT IS FOR GUIDANCE ONLY. IF USED AS PART OF A REVIEW PROCEDURE FOR PUBLICATION, IT SHOULD NOT REPLACE THE EXPERTISE OF AN EXPERIENCED CRYSTALLOGRAPHIC REFEREE.

No syntax errors found.   CIF dictionary   Interpreting this report

Datablock: k09188

Bond precision:	C-C = 0.0042 A		Wavelength=0.71073
Cell:	a=20.5793(6)	b=8.1384(3)	c=17.5927(6)
	alpha=90	beta=113.6580(18)	gamma=90
Temperature:	150 K		

	Calculated	Reported
Volume	2698.84(16)	2698.84(16)
Space group	C 2/c	C 2/c
Hall group	-C 2yc	-C 2yc
Moiety formula	C26 H26 Mo N4 O10	C26 H26 Mo N4 O10
Sum formula	C26 H26 Mo N4 O10	C26 H26 Mo N4 O10
Mr	650.45	650.45
Dx,g cm-3	1.601	1.601
Z	4	4
Mu (mm-1)	0.551	0.551
F000	1328.0	1328.0
F000'	1321.76	
h,k,lmax	26,10,22	26,10,22
Nref	3110	3089
Tmin,Tmax	0.896,0.916	0.851,0.917
Tmin'	0.896	

Correction method= # Reported T Limits: Tmin=0.851 Tmax=0.917
AbsCorr = MULTI-SCAN

Data completeness= 0.993   Theta(max)= 27.509

R(reflections)= 0.0404(2603)   wR2(reflections)= 0.1022(3089)

S = 1.107   Npar= 188

```
The following ALERTS were generated. Each ALERT has the format
       test-name_ALERT_alert-type_alert-level.
Click on the hyperlinks for more details of the test.
```

Alert level C

```
PLAT094_ALERT_2_C Ratio of Maximum / Minimum Residual Density ....       2.20 Report
PLAT971_ALERT_2_C Check Calcd Resid. Dens.  2.24Ang From C7              1.60 eA-3
```

Alert level G

```
PLAT083_ALERT_2_G SHELXL Second Parameter in WGHT  Unusually Large       5.36 Why ?
PLAT398_ALERT_2_G Deviating  C-O-C    Angle From 120 for O3             107.5 Degree
PLAT794_ALERT_5_G Tentative Bond Valency for Mo1       (VI)      .       6.04 Info
PLAT910_ALERT_3_G Missing # of FCF Reflection(s) Below Theta(Min).          2 Note
PLAT912_ALERT_4_G Missing # of FCF Reflections Above STh/L=  0.600         18 Note
PLAT941_ALERT_3_G Average HKL Measurement Multiplicity ..........         4.2 Low
PLAT978_ALERT_2_G Number C-C Bonds with Positive Residual Density.          3 Info
```

```
   0 ALERT level A = Most likely a serious problem - resolve or explain
   0 ALERT level B = A potentially serious problem, consider carefully
   2 ALERT level C = Check. Ensure it is not caused by an omission or oversight
   7 ALERT level G = General information/check it is not something unexpected

   0 ALERT type 1 CIF construction/syntax error, inconsistent or missing data
   5 ALERT type 2 Indicator that the structure model may be wrong or deficient
   2 ALERT type 3 Indicator that the structure quality may be low
   1 ALERT type 4 Improvement, methodology, query or suggestion
   1 ALERT type 5 Informative message, check
```

Validation response form

Please find below a validation response form (VRF) that can be filled in and pasted into your CIF.

```
# start Validation Reply Form
_vrf_PLAT094_k09188
;
PROBLEM: Ratio of Maximum / Minimum Residual Density ....       2.20 Report
RESPONSE: ...
;
_vrf_PLAT971_k09188
;
PROBLEM: Check Calcd Resid. Dens.  2.24Ang From C7              1.60 eA-3
RESPONSE: ...
;
# end Validation Reply Form
```

It is advisable to attempt to resolve as many as possible of the alerts in all categories. Often the minor alerts point to easily fixed oversights, errors and omissions in your CIF or refinement strategy, so attention to these fine details can be worthwhile. In order to resolve some of the more serious problems it may be necessary to carry out additional measurements or structure refinements. However, the purpose of your study may justify the reported deviations and the more serious of these should normally be commented upon in the discussion or experimental section of a paper or in the "special_details" fields of the CIF. checkCIF was carefully designed to identify outliers and unusual parameters, but every test has its limitations and alerts that are not important in a particular case may appear. Conversely, the absence of alerts does not guarantee there are no aspects of the results needing attention. It is up to the individual to critically assess their own results and, if necessary, seek expert advice.

Publication of your CIF in IUCr journals

A basic structural check has been run on your CIF. These basic checks will be run on all CIFs submitted for publication in IUCr journals (*Acta Crystallographica*, *Journal of Applied Crystallography*, *Journal of Synchrotron Radiation*); however, if you intend to submit to *Acta Crystallographica Section C* or *E* or *IUCrData*, you should make sure that full publication checks are run on the final version of your CIF prior to submission.

Publication of your CIF in other journals

Please refer to the *Notes for Authors* of the relevant journal for any special instructions relating to CIF submission.

PLATON version of 18/12/2021; check.def file version of 18/12/2021

Datablock k09188 - ellipsoid plot

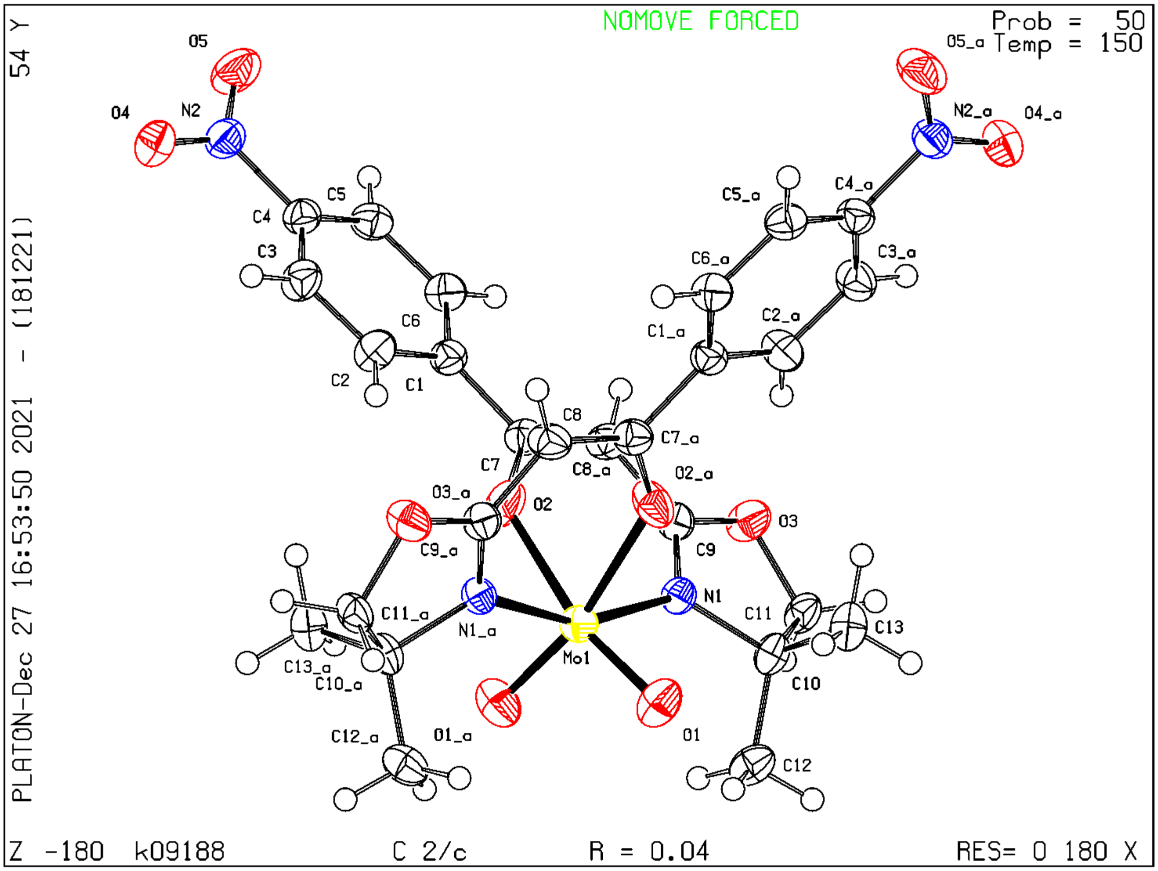

Supplement: Supplementary file 1 [file molecules-27-01309-s001.zip › molecules-1551548-supplementary/ESI-zipped/checkcif_2131175.pdf]
