# Supplementary material for: Synthesis, Characterization, and Catalytic Exploration of Mononuclear Mo(VI) Dioxido Complexes of (Z)-1-R-2-(4′,4′-Dimethyl-2′-oxazolin-2′-yl)-eth-1-en-1-ates
Source: Molecules. 2022 Feb 15;27(4):1309. doi: 10.3390/molecules27041309 (PMC8874378; doi:10.3390/molecules27041309)
Supplement: Supplementary file 1 [file molecules-27-01309-s001.zip › molecules-1551548-supplementary/ESI-zipped/SupplementaryInformation.pdf]

Supplementary Information for:

Synthesis, Characterisation and Catalytic Exploration of Mononuclear Mo(VI) Dioxido Complexes of the (Z)-1-R-2-(4',4'-dimethyl-2'-oxazolin-2'-yl)-eth-1-en-1-ates

Authors: A. Petrov, J. A. Adjei, A. J. Lough, R. S. Wylie & R. A. Gossage

UV-Visible spectroscopy of Complexes **1**-**4** ………………………………………………… S-2

Physical Appearances of Complexes **1**-**4** …………………………………………………… S-7

IR spectra of **1**-**4** ……………………………………………………………………………….. S-8

Mo(O)$_2$(L^n)$_2$ complexes – IR and UV-Vis.

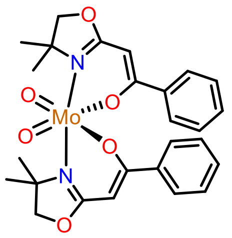

Molecular Weight: 560.4690

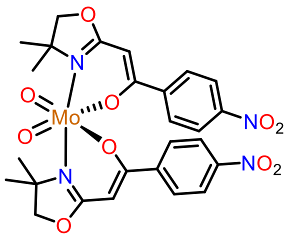

Molecular Weight: 650.4630

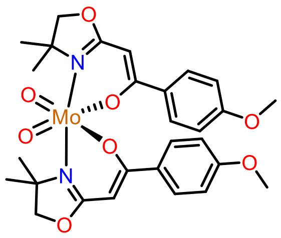

Molecular Weight: 620.5210

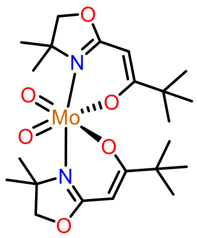

Molecular Weight: 520.4890

All together – normalized absorbance

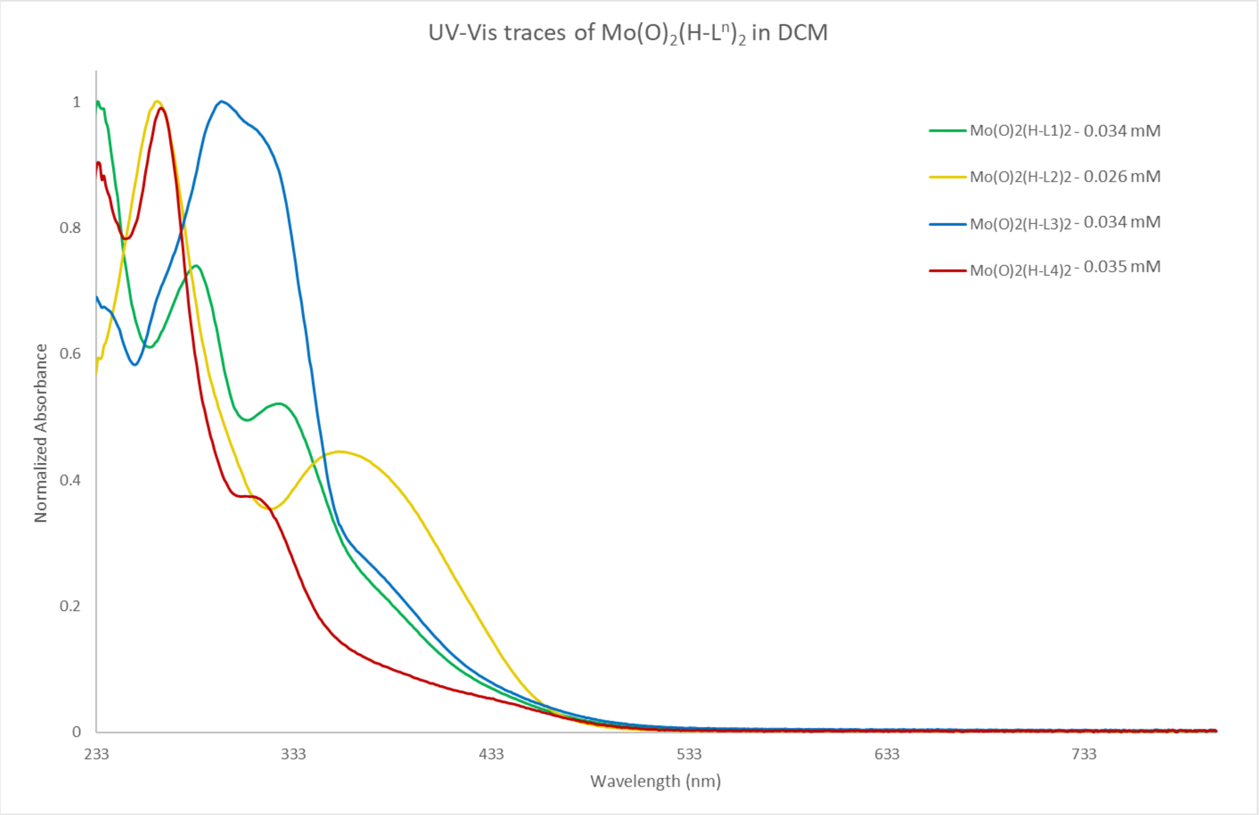

Graph S1: UV-Visible traces of all Mo complexes taken in DCM. Absorbance is normalized for clarity.

Table S1: Selected UV-Vis data for all four complexes analyzed. Each complex was dissolved in DCM and diluted to an approximate concentration of 0.03 mM. Absorbances shown in table are not normalized but instead is the raw unaltered data.

Complex	Conc. (mM)	λ_{max} (nm)			Absorbance			Molar Absorptivity (L/cm·mol)		
		Max$_1$	Max$_2$	Max$_3$	Abs$_1$	Abs$_2$	Abs$_3$	ε_1	ε_2	ε_3
Mo(O)$_2$(H-L^1)$_2$	0.0345	284	326	393	0.903	0.636	0.207	26159	18427	5998
Mo(O)$_2$(H-L^2)$_2$	0.0263	266	258	-	0.968	0.432	-	36818	16420	-
Mo(O)$_2$(H-L^3)$_2$	0.0343	297	313	378	1.129	1.081	0.280	32906	91526	8150
Mo(O)$_2$(H-L^4)$_2$	0.0354	266	308	434	0.674	0.254	0.035	19048	7171	987

Complex **1**: $Mo(O)_2(L^1)_2$

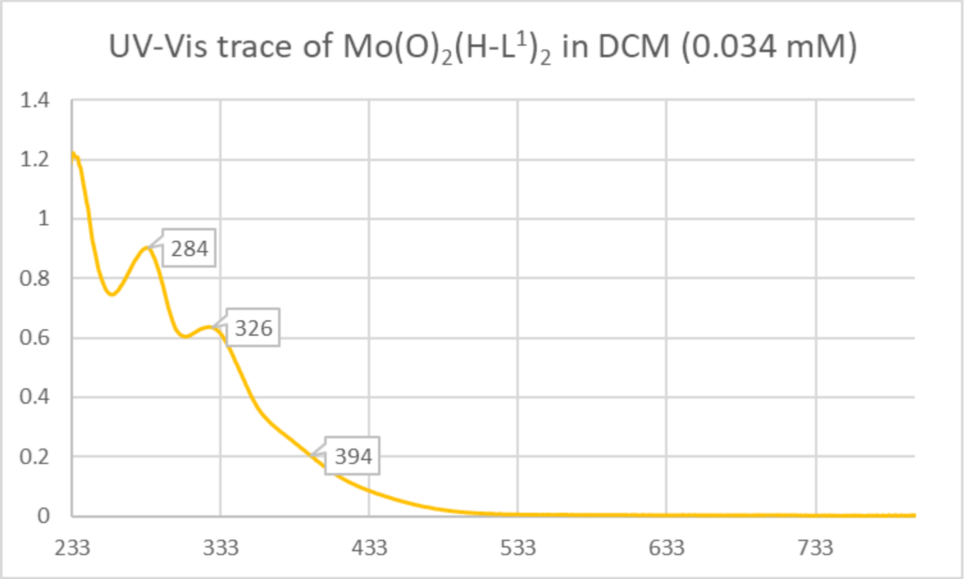

Graph S2: UV-Vis trace of $Mo(O)_2(L^1)_2$ in DCM (0.0345 mM). Absorbance is not normalized to 1.

Complex **2**: $Mo(O)_2(L^2)_2$

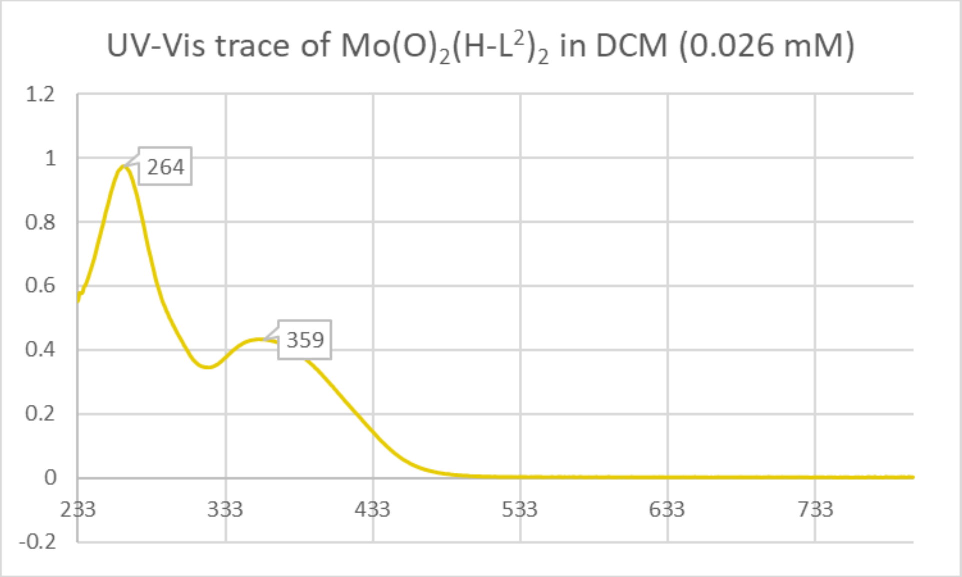

Graph S3: UV-Vis trace of $Mo(O)_2(L^2)_2$ in DCM (0.0263 mM). Absorbance is not normalized to 1.

Complex **3**: $Mo(O)_2(L^3)_2$

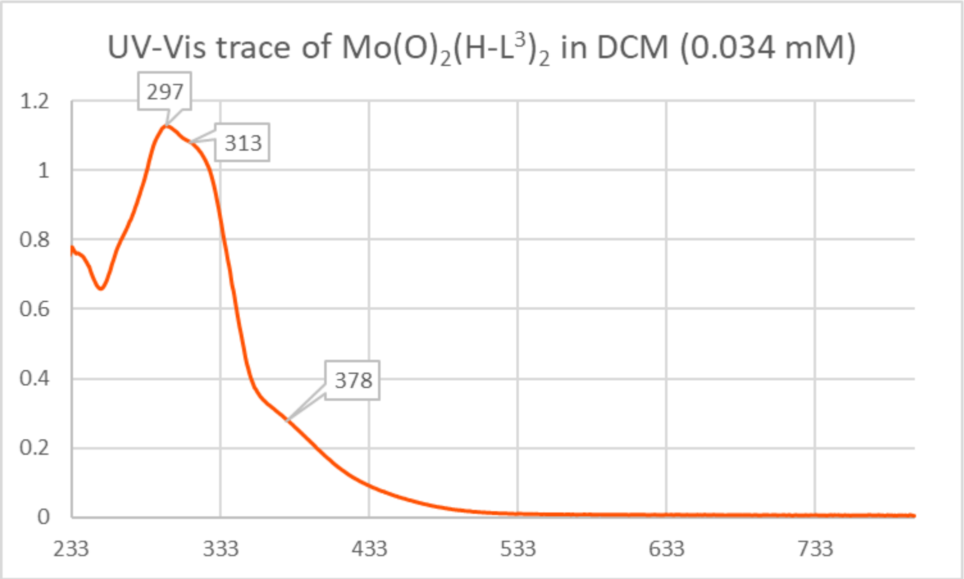

Graph S4: UV-Vis trace of $Mo(O)_2(L^3)_2$ in DCM (0.0343 mM). Absorbance is not normalized to 1.

Complex **4**: $Mo(O)_2(L^4)_2$

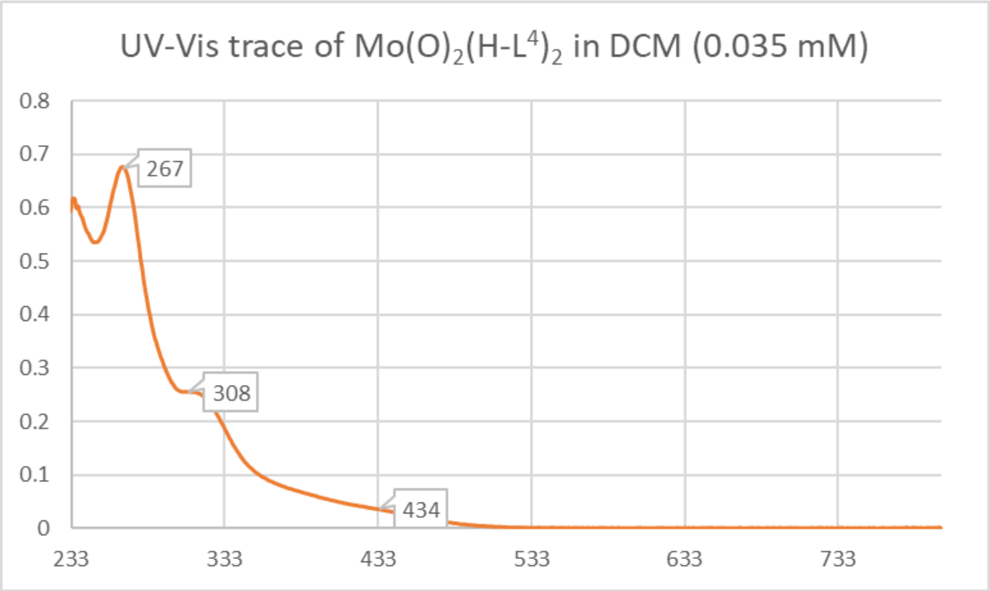

Graph S5: UV-Vis trace of $Mo(O)_2(L^4)_2$ in DCM (0.0354 mM). Absorbance is not normalized to 1.

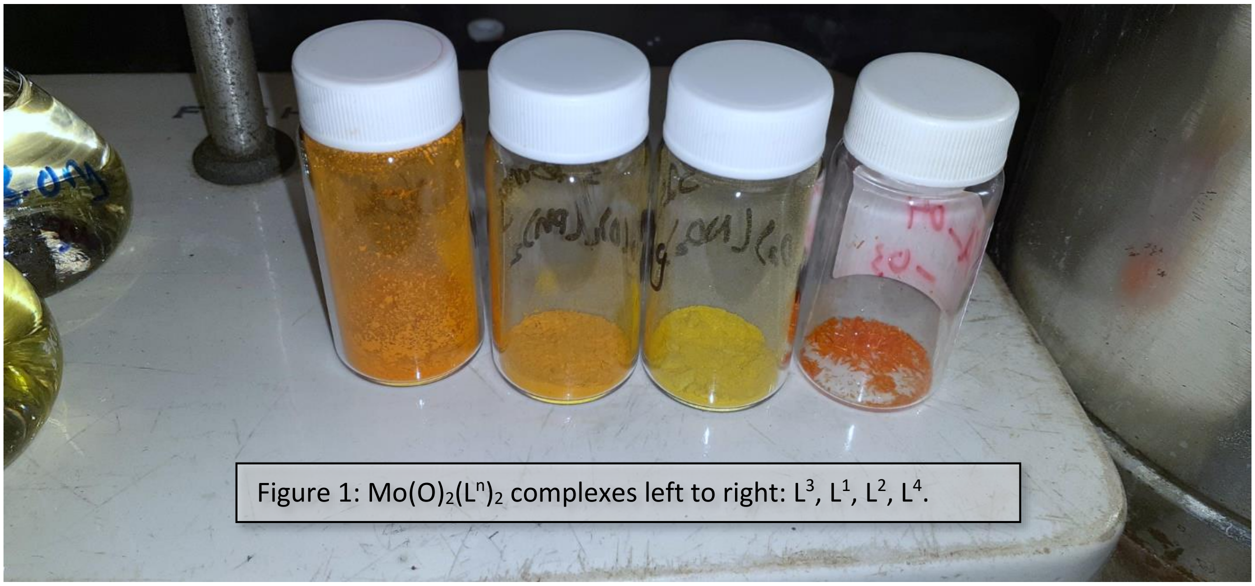

Figure 1: $Mo(O)_2(L^n)_2$ complexes left to right: L^3, L^1, L^2, L^4.

Physical appearances of complexes **1**-**4**.

Complex **1**

Mo(O)$_2$(L^1)$_2$

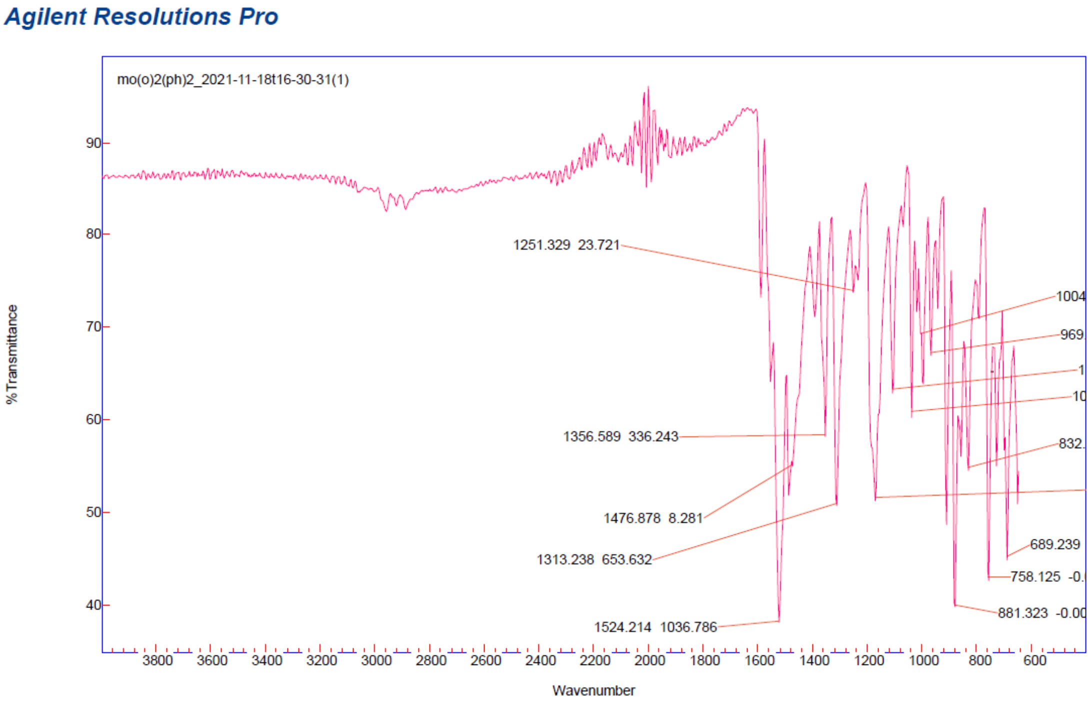

Graph S6: IR spectrum of Mo(O)$_2$(L^1)$_2$. ν C=N: 1524, Mo=O: 881, Mo=O: 961, Mo-O: 758.

Complex **2**:

$Mo(O)_2(L^2)_2$

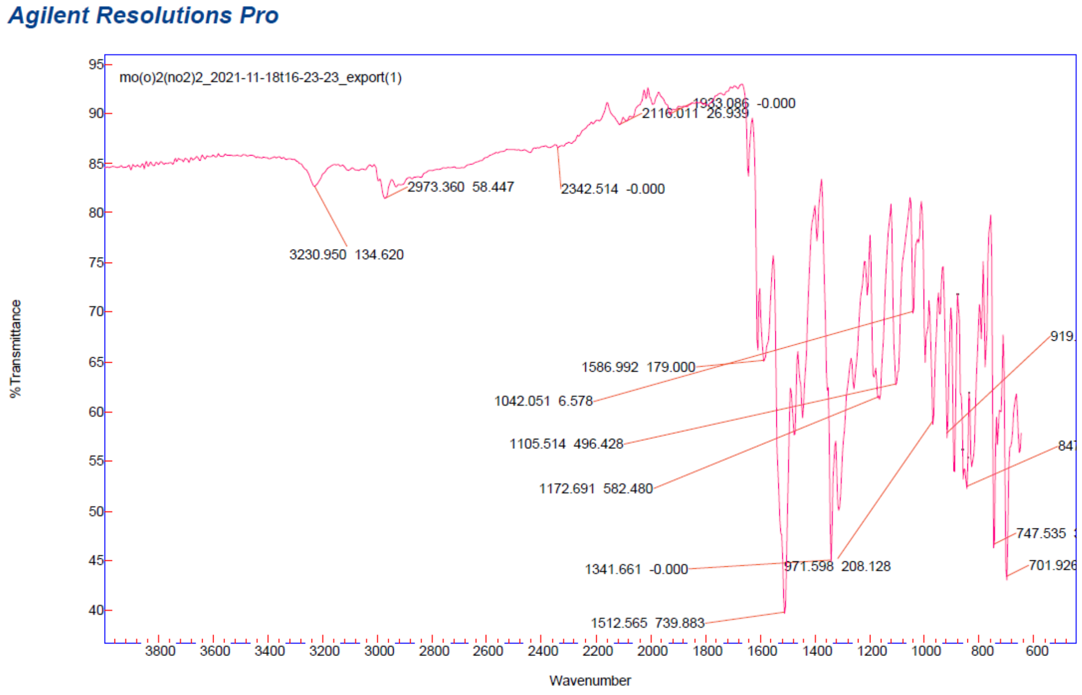

Graph S7: IR spectrum of $Mo(O)_2(L^2)_2$. ν C=N: 1512, Mo=O: 971, Mo-O: 747.

Complex **3**:

$Mo(O)_2(L^3)_2$

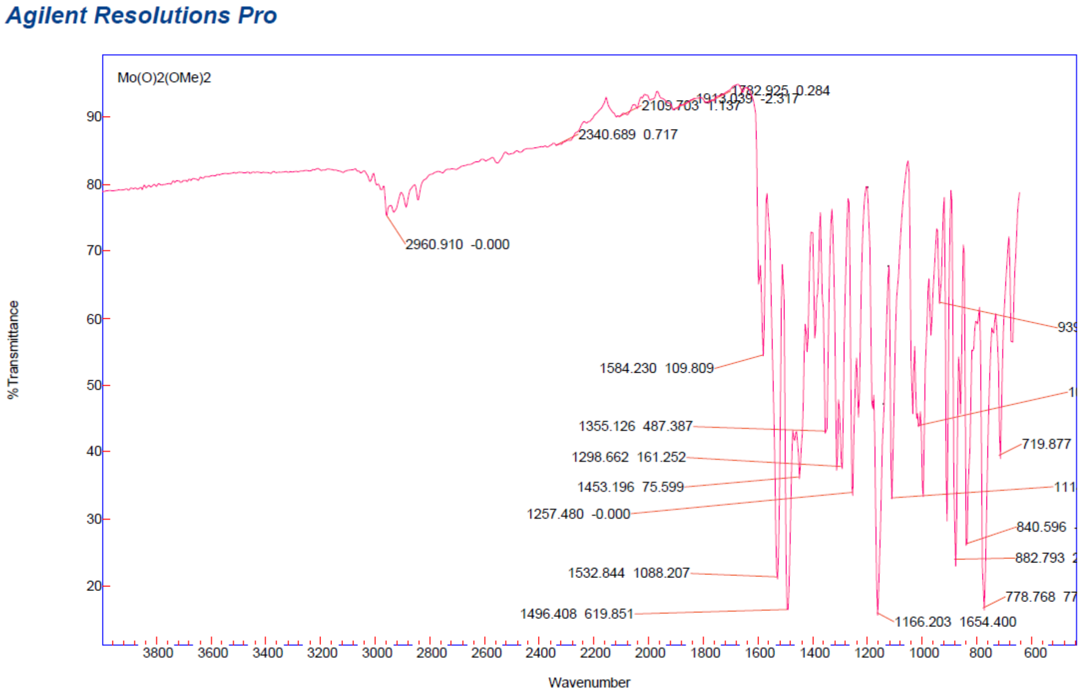

Graph S8: IR spectrum of $Mo(O)_2(H\text{-}L^3)_2$. ν C=N: 1532, Mo=O: 882, Mo-O: 778.

Complex **4**:

$Mo(O)_2(L^4)_2$

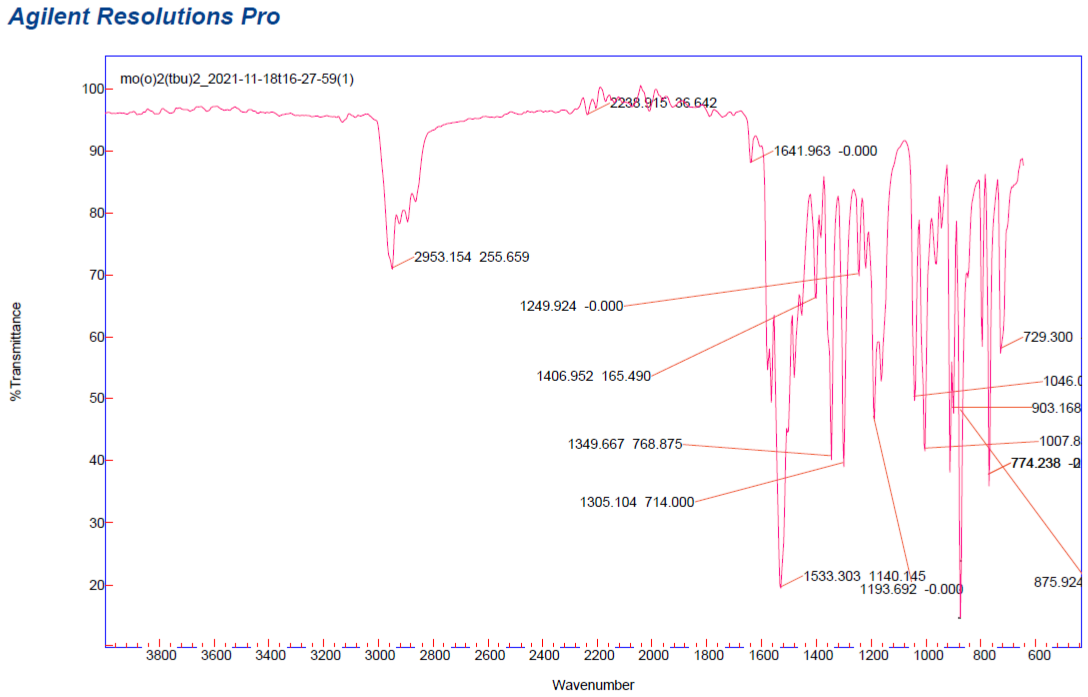

Graph S9: IR spectrum of $Mo(O)_2(L^4)_2$. ν C=N: 1533, Mo=O: 850. Mo-O: 774.
