# Supplementary material for: Synthesis, Characterization, and Catalytic Exploration of Mononuclear Mo(VI) Dioxido Complexes of (Z)-1-R-2-(4′,4′-Dimethyl-2′-oxazolin-2′-yl)-eth-1-en-1-ates
Source: Molecules. 2022 Feb 15;27(4):1309. doi: 10.3390/molecules27041309 (PMC8874378; doi:10.3390/molecules27041309)

checkCIF/PLATON report

Structure factors have been supplied for datablock(s) k1048

THIS REPORT IS FOR GUIDANCE ONLY. IF USED AS PART OF A REVIEW PROCEDURE FOR PUBLICATION, IT SHOULD NOT REPLACE THE EXPERTISE OF AN EXPERIENCED CRYSTALLOGRAPHIC REFEREE.

No syntax errors found. CIF dictionary Interpreting this report

Datablock: k1048

Bond precision:	C-C = 0.0061 A		Wavelength=0.71073
Cell:	a=19.9342(10)	b=19.9342(10)	c=11.5030(4)
	alpha=90	beta=90	gamma=120
Temperature:	150 K		

	Calculated	Reported
Volume	3958.6(4)	3958.6(4)
Space group	P -3 c 1	P -3 c 1
Hall group	-P 3 2"c	-P 3 2"c
Moiety formula	C22 H36 Mo N2 O6	C22 H36 Mo N2 O6
Sum formula	C22 H36 Mo N2 O6	C22 H36 Mo N2 O6
Mr	520.47	520.47
Dx,g cm-3	1.310	1.310
Z	6	6
Mu (mm-1)	0.532	0.532
F000	1632.0	1632.0
F000’	1622.33	
h,k,lmax	25,25,14	22,25,14
Nref	3035	3021
Tmin,Tmax	0.899,0.948	0.897,0.949
Tmin’	0.899	

Correction method= # Reported T Limits: Tmin=0.897 Tmax=0.949
AbsCorr = MULTI-SCAN

Data completeness= 0.995 Theta(max)= 27.479

R(reflections)= 0.0522(2090) wR2(reflections)= 0.1511(3021)

S = 1.041 Npar= 146

The following ALERTS were generated. Each ALERT has the format
test-name_ALERT_alert-type_alert-level.
Click on the hyperlinks for more details of the test.

Alert level A

PLAT601_ALERT_2_A Unit Cell Contains Solvent Accessible VOIDS of . 329 Ang**3

Author Response: During the refinement of the structure, electron density peaks were located that were believed to be highly disordered solvent molecules (possibly chlorform/hexane/acetone). Attempts made to model the solvent molecule were not successful. The SQUEEZE (Spek, 2015) option in PLATON (Spek, 2009) indicated there was a solvent cavity of volume 329 \%A^3^ containing approximately 76 electrons. In the final cycles of refinement, this contribution to the electron density was removed from the observed data. The density, the F(000) value, the molecular weight and the formula are given without taking into account the results obtained with the SQUEEZE option PLATON. Similar treatments of disordered solvent molecules were carried out in the recent structure determination given below. References: Spek, A. L. (2015). Acta Cryst. C71, 9--18. Spek, A. L. (2009). *Acta Cryst.* D65, 148--155. R. Manohar, M. Harikrishna, S. H. Etti, C. Ramanathan and K. Gunasekaran Acta Cryst. (2019). E75, 562-564 R. Vishnupriya, M. Venkateshan, J. Suresh, R. V. Sumesh, R. R. Kumar and P. L. N. Lakshman Acta Cryst. (2019). E75, 189-193 K. B. Polyanskii, K. A. Alekseeva, P. A. Kumandin, Z. Atioglu, M. Akkurt and F. A. A. Toze Acta Cryst. (2019). E75, 342-345 T. I. Ayudhya, A. L. Rheingold and N. N. Dingra Acta Cryst. (2019). E75, 543-546 S. Syed Abuthahir, M. NizamMohideen, V. Viswanathan, T. Abiraman and S. Balasubramanian Acta Cryst. (2019). E75, 655-661

Alert level C

```
PLAT241_ALERT_2_C High   'MainMol' Ueq as Compared to Neighbors of         O1 Check
PLAT241_ALERT_2_C High   'MainMol' Ueq as Compared to Neighbors of         C8 Check
PLAT242_ALERT_2_C Low    'MainMol' Ueq as Compared to Neighbors of        Mo1 Check
PLAT242_ALERT_2_C Low    'MainMol' Ueq as Compared to Neighbors of         C2 Check
PLAT242_ALERT_2_C Low    'MainMol' Ueq as Compared to Neighbors of         C9 Check
PLAT250_ALERT_2_C Large U3/U1 Ratio for Average U(i,j) Tensor ....        2.2 Note
PLAT906_ALERT_3_C Large K Value in the Analysis of Variance ......      4.973 Check
PLAT918_ALERT_3_C Reflection(s) with I(obs) much Smaller I(calc) .          1 Check
PLAT973_ALERT_2_C Check Calcd Positive Resid. Density on       Mo1       1.17 eA-3
```

Alert level G

```
PLAT066_ALERT_1_G Predicted and Reported Tmin&Tmax Range Identical          ? Check
PLAT398_ALERT_2_G Deviating  C-O-C    Angle From 120 for O2             107.3 Degree
PLAT794_ALERT_5_G Tentative Bond Valency for Mo1       (VI)      .       6.19 Info
PLAT883_ALERT_1_G No Info/Value for _atom_sites_solution_primary .     Please Do !
PLAT910_ALERT_3_G Missing # of FCF Reflection(s) Below Theta(Min).          3 Note
PLAT912_ALERT_4_G Missing # of FCF Reflections Above STh/L=  0.600         10 Note
PLAT913_ALERT_3_G Missing # of Very Strong Reflections in FCF ....          2 Note
PLAT941_ALERT_3_G Average HKL Measurement Multiplicity ..........         1.0 Low
```

```
PLAT950_ALERT_5_G Calculated (ThMax) and CIF-Reported Hmax Differ          3 Units
PLAT961_ALERT_5_G Dataset Contains no Negative Intensities .......     Please Check
PLAT978_ALERT_2_G Number C-C Bonds with Positive Residual Density.         0 Info
```

```
   1 ALERT level A = Most likely a serious problem - resolve or explain
   0 ALERT level B = A potentially serious problem, consider carefully
   9 ALERT level C = Check. Ensure it is not caused by an omission or oversight
  11 ALERT level G = General information/check it is not something unexpected

   2 ALERT type 1 CIF construction/syntax error, inconsistent or missing data
  10 ALERT type 2 Indicator that the structure model may be wrong or deficient
   5 ALERT type 3 Indicator that the structure quality may be low
   1 ALERT type 4 Improvement, methodology, query or suggestion
   3 ALERT type 5 Informative message, check
```

It is advisable to attempt to resolve as many as possible of the alerts in all categories. Often the minor alerts point to easily fixed oversights, errors and omissions in your CIF or refinement strategy, so attention to these fine details can be worthwhile. In order to resolve some of the more serious problems it may be necessary to carry out additional measurements or structure refinements. However, the purpose of your study may justify the reported deviations and the more serious of these should normally be commented upon in the discussion or experimental section of a paper or in the "special_details" fields of the CIF. checkCIF was carefully designed to identify outliers and unusual parameters, but every test has its limitations and alerts that are not important in a particular case may appear. Conversely, the absence of alerts does not guarantee there are no aspects of the results needing attention. It is up to the individual to critically assess their own results and, if necessary, seek expert advice.

Publication of your CIF in IUCr journals

A basic structural check has been run on your CIF. These basic checks will be run on all CIFs submitted for publication in IUCr journals (*Acta Crystallographica*, *Journal of Applied Crystallography*, *Journal of Synchrotron Radiation*); however, if you intend to submit to *Acta Crystallographica Section C* or *E* or *IUCrData*, you should make sure that full publication checks are run on the final version of your CIF prior to submission.

Publication of your CIF in other journals

Please refer to the *Notes for Authors* of the relevant journal for any special instructions relating to CIF submission.

Validation response form

Please find below a validation response form (VRF) that can be filled in and pasted into your CIF.

```
# start Validation Reply Form
_vrf_PLAT241_k1048
;
PROBLEM: High   'MainMol' Ueq as Compared to Neighbors of        O1 Check
RESPONSE: ...
```

```
;
_vrf_PLAT242_k1048
;
PROBLEM: Low    'MainMol' Ueq as Compared to Neighbors of        Mo1 Check
RESPONSE: ...
;
_vrf_PLAT250_k1048
;
PROBLEM: Large U3/U1 Ratio for Average U(i,j) Tensor ....        2.2 Note
RESPONSE: ...
;
_vrf_PLAT906_k1048
;
PROBLEM: Large K Value in the Analysis of Variance ......      4.973 Check
RESPONSE: ...
;
_vrf_PLAT918_k1048
;
PROBLEM: Reflection(s) with I(obs) much Smaller I(calc) .          1 Check
RESPONSE: ...
;
_vrf_PLAT973_k1048
;
PROBLEM: Check Calcd Positive Resid. Density on       Mo1       1.17 eA-3
RESPONSE: ...
;
# end Validation Reply Form
```

PLATON version of 18/12/2021; check.def file version of 18/12/2021

Datablock k1048 - ellipsoid plot

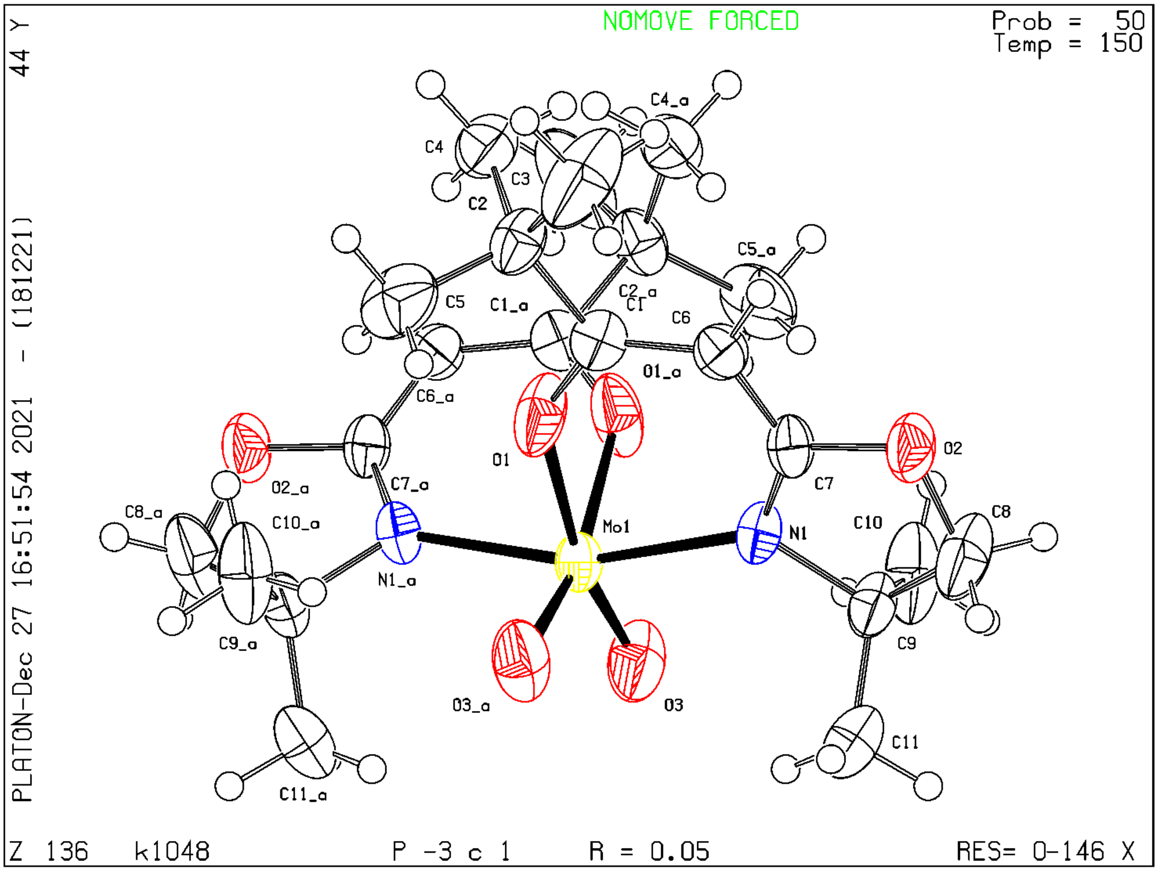

Supplement: Supplementary file 1 [file checkcif_2131174.pdf › molecules-1551548-supplementary/ESI-zipped/checkcif_2131174.pdf]
