# Supplementary material for: Synthesis, Characterization, and Catalytic Exploration of Mononuclear Mo(VI) Dioxido Complexes of (Z)-1-R-2-(4′,4′-Dimethyl-2′-oxazolin-2′-yl)-eth-1-en-1-ates
Source: Molecules. 2022 Feb 15;27(4):1309. doi: 10.3390/molecules27041309 (PMC8874378; doi:10.3390/molecules27041309)

checkCIF/PLATON report

Structure factors have been supplied for datablock(s) k1044

THIS REPORT IS FOR GUIDANCE ONLY. IF USED AS PART OF A REVIEW PROCEDURE FOR PUBLICATION, IT SHOULD NOT REPLACE THE EXPERTISE OF AN EXPERIENCED CRYSTALLOGRAPHIC REFEREE.

No syntax errors found. CIF dictionary Interpreting this report

Datablock: k1044

```
Bond precision:   C-C = 0.0046 A           Wavelength=0.71073

Cell:              a=11.2202(2)     b=12.9081(3)     c=18.7510(4)
                   alpha=90         beta=90          gamma=90
Temperature:       150 K

                Calculated                    Reported
Volume          2715.74(10)                   2715.73(10)
Space group     P b c n                       P b c n
Hall group      -P 2n 2ab                     -P 2n 2ab
Moiety formula  C28 H32 Mo N2 O8              C28 H32 Mo N2 O8
Sum formula     C28 H32 Mo N2 O8              C28 H32 Mo N2 O8
Mr              620.50                        620.49
Dx,g cm-3       1.518                         1.518
Z               4                             4
Mu (mm-1)       0.536                         0.536
F000            1280.0                        1280.0
F000'           1273.69
h,k,lmax        14,16,24                      14,16,24
Nref            3123                          3120
Tmin,Tmax       0.914,0.938                   0.629,0.770
Tmin'           0.908

Correction method= # Reported T Limits: Tmin=0.629 Tmax=0.770
AbsCorr = MULTI-SCAN

Data completeness= 0.999           Theta(max)= 27.484

R(reflections)= 0.0449( 2144)                          wR2(reflections)=
                                                       0.1339( 3120)
S = 1.071                  Npar= 183
```

The following ALERTS were generated. Each ALERT has the format
test-name_ALERT_alert-type_alert-level.
Click on the hyperlinks for more details of the test.

Alert level C

```
PLAT241_ALERT_2_C High   'MainMol' Ueq as Compared to Neighbors of         O1 Check
PLAT241_ALERT_2_C High   'MainMol' Ueq as Compared to Neighbors of        C11 Check
PLAT905_ALERT_3_C Negative K value in the Analysis of Variance ...     -0.695 Report
```

Alert level G

```
PLAT004_ALERT_5_G Polymeric Structure Found with Maximum Dimension          1 Info
PLAT301_ALERT_3_G Main Residue  Disorder ..............(Resd  1  )         5% Note
```

Author Response: In the final cycles of refinement, one peak which was significantly larger (ca. 2 eA3) was refined as a minor component of disorder from Mo1. This may be attributed to whole molecule disorder where only the minor component related to the Mo atom can be identified as the contribution is so small. This refinment works well and prevent the presence of an A-alert.**

```
PLAT303_ALERT_2_G Full Occupancy Atom H11A      with # Connections       1.03 Check
PLAT367_ALERT_2_G Long?  C(sp?)-C(sp?) Bond  C11      - C12      .       1.53 Ang.
PLAT398_ALERT_2_G Deviating  C-O-C    Angle From 120 for O2             106.7 Degree
PLAT779_ALERT_4_G Suspect or Irrelevant (Bond) Angle(s) in CIF ...      25.40 Deg.
              MO1A -C11  -H11A    1_555   1_555   1_555 ......     #   63 Check
PLAT910_ALERT_3_G Missing # of FCF Reflection(s) Below Theta(Min).          2 Note
PLAT912_ALERT_4_G Missing # of FCF Reflections Above STh/L=  0.600          1 Note
PLAT978_ALERT_2_G Number C-C Bonds with Positive Residual Density.          0 Info
```

0 **ALERT level A** = Most likely a serious problem - resolve or explain
0 **ALERT level B** = A potentially serious problem, consider carefully
3 **ALERT level C** = Check. Ensure it is not caused by an omission or oversight
9 **ALERT level G** = General information/check it is not something unexpected

0 ALERT type 1 CIF construction/syntax error, inconsistent or missing data
6 ALERT type 2 Indicator that the structure model may be wrong or deficient
3 ALERT type 3 Indicator that the structure quality may be low
2 ALERT type 4 Improvement, methodology, query or suggestion
1 ALERT type 5 Informative message, check

Validation response form

Please find below a validation response form (VRF) that can be filled in and pasted into your CIF.

```
# start Validation Reply Form
_vrf_PLAT241_k1044
;
PROBLEM: High   'MainMol' Ueq as Compared to Neighbors of         O1 Check
RESPONSE: ...
;
```

```
_vrf_PLAT905_k1044
;
PROBLEM: Negative K value in the Analysis of Variance ...     -0.695 Report
RESPONSE: ...
;
# end Validation Reply Form
```

It is advisable to attempt to resolve as many as possible of the alerts in all categories. Often the minor alerts point to easily fixed oversights, errors and omissions in your CIF or refinement strategy, so attention to these fine details can be worthwhile. In order to resolve some of the more serious problems it may be necessary to carry out additional measurements or structure refinements. However, the purpose of your study may justify the reported deviations and the more serious of these should normally be commented upon in the discussion or experimental section of a paper or in the "special_details" fields of the CIF. checkCIF was carefully designed to identify outliers and unusual parameters, but every test has its limitations and alerts that are not important in a particular case may appear. Conversely, the absence of alerts does not guarantee there are no aspects of the results needing attention. It is up to the individual to critically assess their own results and, if necessary, seek expert advice.

Publication of your CIF in IUCr journals

A basic structural check has been run on your CIF. These basic checks will be run on all CIFs submitted for publication in IUCr journals (*Acta Crystallographica*, *Journal of Applied Crystallography*, *Journal of Synchrotron Radiation*); however, if you intend to submit to *Acta Crystallographica Section C* or *E* or *IUCrData*, you should make sure that full publication checks are run on the final version of your CIF prior to submission.

Publication of your CIF in other journals

Please refer to the *Notes for Authors* of the relevant journal for any special instructions relating to CIF submission.

PLATON version of 18/12/2021; check.def file version of 18/12/2021

Datablock k1044 - ellipsoid plot

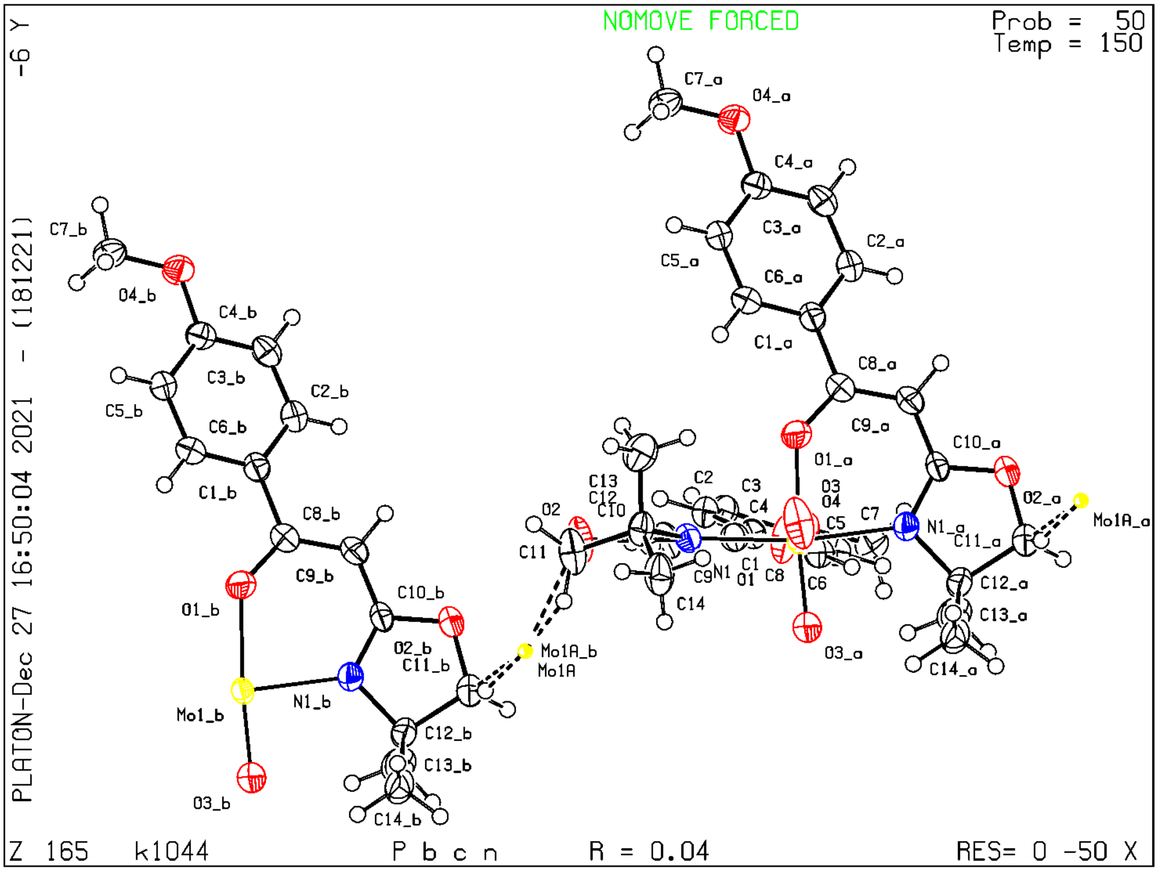

Supplement: Supplementary file 1 [file checkcif_2131176.pdf › molecules-1551548-supplementary/ESI-zipped/checkcif_2131176.pdf]
